# Comparative assessment of the stability of buccal shelf mini-screws with and without pre-drilling- a split-mouth, randomized controlled trial

**DOI:** 10.1007/s00784-024-05925-7

**Published:** 2024-10-04

**Authors:** Arshia Ummat, Siddarth Shetty, Asavari Desai, Supriya Nambiar, Srikant Natarajan

**Affiliations:** 1 Department of Orthodontics, Manipal College of Dental Sciences Mangalore, Manipal Academy of Higher Education, Manipal Karnataka, 576104 India; 2 Department of Oral Pathology, Manipal College of Dental Sciences Mangalore, Manipal Academy of Higher Education, Manipal Karnataka, 576104 India

**Keywords:** Buccal shelf bone screws, Primary stability, Secondary stability, Class III camouflage

## Abstract

**Objectives:**

To examine and compare the stability of buccal shelf mini-screws using self-drilling and pre-drilling implant placement techniques.

**Methodology:**

It was a split-mouth, randomized controlled trial comprising of 7 patients, each receiving two buccal shelf bone screws. The screws were placed using a self-drilling protocol in one quadrant and a pre-drilling protocol in the opposing quadrant decided via coin toss randomization. Stability was examined at the time of placement and 1,2, 3 and 4 months thereafter, using the Resonance Frequency Analysis method with the Osstell Beacon ^TM^ device. The Implant Stability Quotient (ISQ) obtained was then compared and assessed between both quadrants. Friedman’s Two-Way Analysis of Variance and the Wilcoxon signed rank test were utilized for the intergroup comparison. A statistically significant result was defined as one with a p-value of less than 0.05.

**Results:**

A statistically significant difference between the mean ISQ reading in the pre-drilling and self-drilling group was observed, indicating higher stability of bone screws placed with the pre-drilling protocol. The primary stability of the buccal shelf screws decreased after placement, but the secondary stability remained stable.

**Conclusion:**

Buccal shelf bone screws placed with a pre-drilling protocol depicted greater primary and secondary stability as compared to the self-drilling protocol, as depicted by the ISQ readings obtained. Resonance Frequency Analysis can be used as a valuable tool to assess the stability of buccal shelf bone screws.

**Clinical relevance:**

The use of buccal shelf screws has increased tremendously over the past few years due to their myriad applications and have now become an essential part of an orthodontist’s armamentarium. It is therefore essential for clinicians to be well-informed about all aspects of their use including insertion techniques. The results of this trial indicate that the pre-drilling protocol provides better stability and therefore treatment outcomes.

## Introduction

Anchorage, which is the resistance to unwanted tooth movement [1] is an essential component of the orthodontic treatment of dental and skeletal malocclusions. Temporary anchorage devices (TADs) have a plethora of applications in clinical orthodontics including anchorage conservation and are an important part of a clinician’s armamentarium.

In recent years, orthodontics has seen significant advancements in TADs with the introduction of infra-zygomatic crest (IZC), and buccal shelf (BS) orthodontic bone screws, leading to a transformation in the field. These innovations have redefined the concept of absolute anchorage, providing orthodontists with versatile tools to tackle complex cases without resorting to surgical interventions.

Buccal shelf screws are indicated for complete arch distalization of the mandibular dentition to conceal a Class III malocclusion as well as for distalization of arches in re-treatment cases of anchorage loss, which are difficult to treat with a standard micro-implant elsewhere [2].

The most preferable location for bone screw insertion in the mandible is the buccal shelf area, which is situated lateral to and just below the region of the second molar [3]. Two types of protocols can be used for insertion: a self-drilling (SD) protocol or a pre-drilling (PD) protocol. In a self-drilling protocol, the bone screw is placed without a punch cut or a pilot drill. The cutting edges of the implant tip are sharp and provide the necessary force for the implant placement. This method may lead to very high insertion torque and in turn an increased resistance to implant placement which in turn can cause a higher amount of bone compression during implant placement.

An alternative method for placing the orthodontic bone screws is with a pre-drilling protocol. This involves making a small punch cut using a tissue punch device, followed by a pilot hole at the desired position and thereafter implant insertion. This reduces bone compression during implant placement and decreases the incidence of implant slippage during the placement protocol.

Several anatomical factors affect both, the primary and secondary stability of the implant - the type of soft tissue (mucosa vs. attached gingiva, tissue thickness, mobility, and proximity to the frenum), the type of bone (bone density, bone depth, cortical bone thickness), and the proximity to particular anatomical structures (roots, nerves, vessels, sinus/nasal cavities) [4].

Primary stability is a mechanical phenomenon and is reliant on the type and quantity of bone present in the area as well as the implant type and placement technique [5, 6]. The formation of new bone and remodelling at the interface between the implant and tissue, as well as in the surrounding bone, are responsible for secondary stability [6].

A meta-analysis carried out by Hong showed that the stability of TADs placed in the mandible is significantly lower than that of the maxilla by 2.23 times. This can be attributed to peri-implant inflammation and irritation from chewing as well as bone characteristics. The dense, thick cortical bone also significantly increases the risk of implant fracture and insertion torque which negatively affects the stability [7].

Clinicians are therefore often faced with the dilemma of choosing the right insertion protocol that ensures maximum stability and minimises the risk of fracture. A literature search revealed that the stability of buccal shelf bone screws placed using the SD and PD protocols has not been previously studied. This trial was designed to assess and compare the outcome of these two methods, which would help orthodontists to make informed decisions.

There are various methods and devices to assess implant stability such as clinical measurement of cutting resistance during implant placement, reverse torque test, and the periotest [8]. These techniques are not reproducible and are often cumbersome to perform. In this study, we decided to employ a new approach called the Resonance Frequency Analysis (RFA) to test for stability. The device consists of a transducer, a metallic rod with a magnet on top, which is attached onto an implant or abutment. A magnetic pulse which has a duration of 1 ms, passing through a wireless probe excites the magnet. After excitation, the peg vibrates freely, and the magnet induces an electric voltage in the probe coil which is measured by the resonance frequency analyzer [9]. The readings recorded indicate the implant stability quotient (ISQ) value. This technique is sensitive, easily reproducible and non-invasive.

### Objectives

To assess and compare the stability of buccal shelf bone screws placed with and without pre-drilling, using Resonance Frequency Analysis (RFA).

## Materials and methods

The study was carried out at the Department of Orthodontics after obtaining approval from the Institutional Ethics Committee (Protocol Ref. no 19099) in accordance with the guidelines laid down by the 1964 Declaration of Helsinki. It was a prospective, split mouth randomized controlled trial and informed consent was obtained from all participants before recruitment.

With an assumption of stability of buccal shelf screws as 85% with pre-drilling and 10% without pre-drilling, 80% power, 95% confidence interval, and 1:1 allocation, a sample size of 14 was arrived at.

The inclusion criteria for the present study were patients between the age group of 18–30 years, requiring fixed orthodontic treatment with the application of buccal shelf screws and with buccal shelf bone thickness of at least 5 mm. Patients presenting with bone pathologies or systemic conditions like diabetes and those with a history of smoking were excluded.

**Fig. 1:**
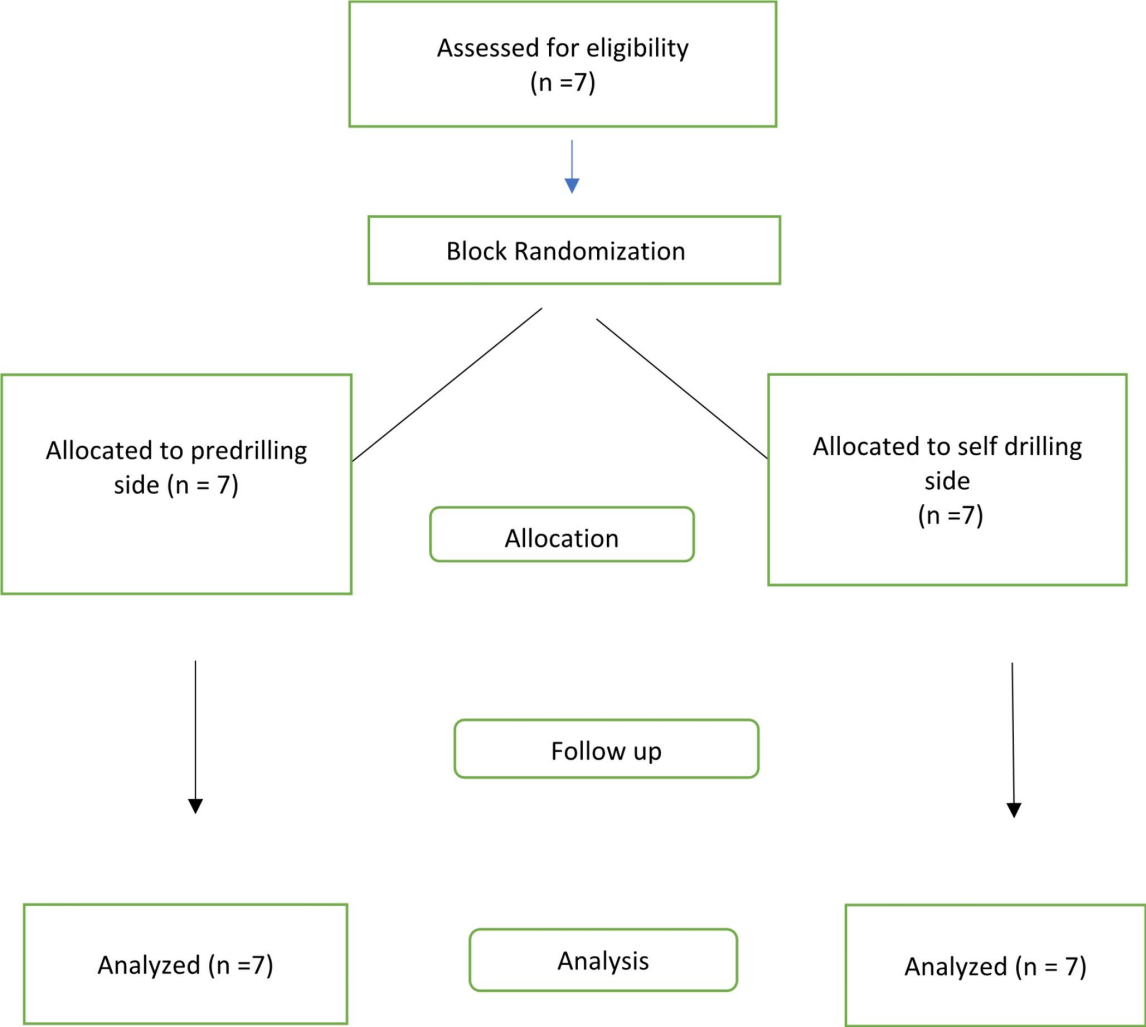
CONSORT chart depicting flow of patients

The recruitment and flow of patients as per the CONSORT guidelines are shown in Fig. 1.

The type of implant that was used is A-1P-212,012 (stainless steel, diameter of 2 mm, length of 12 mm) manufactured by A1 Bio-RayTM Biotech Instrument Co., Ltd. In one quadrant of the mandible, the screw was placed by a self-drilling protocol, and on the other side, the screw was placed after pre-drilling. A 3 mm punch cut was done before pre-drilling to remove the tissue tag.

**Fig. 2:**
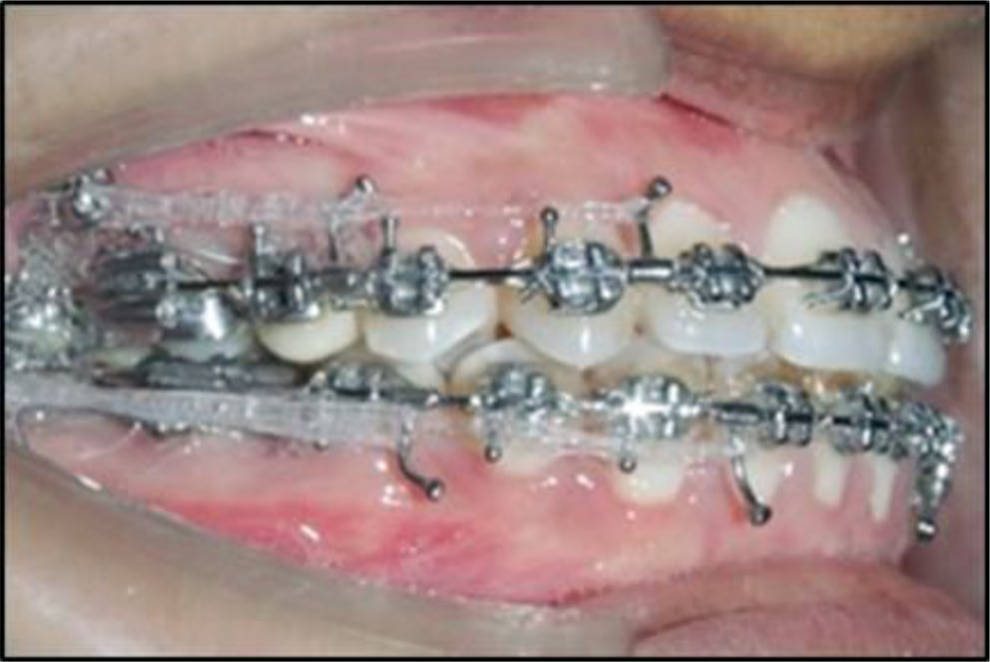
E-chains loaded onto the buccal shelf screws

A standardized force of 400 g was applied to the bone screws with the help of E-chains (3 M Alastik) to effectively aid in the orthodontic procedure. Only one person was responsible for inserting the screws to eliminate inter-operator variability and force was checked using a dontrix gauge to ensure uniformity. Two E-chains were loaded onto the implants, one placed mesial to the canine and the other placed mesial to the pre-molars, as shown in Fig. 2.

**Fig. 3:**
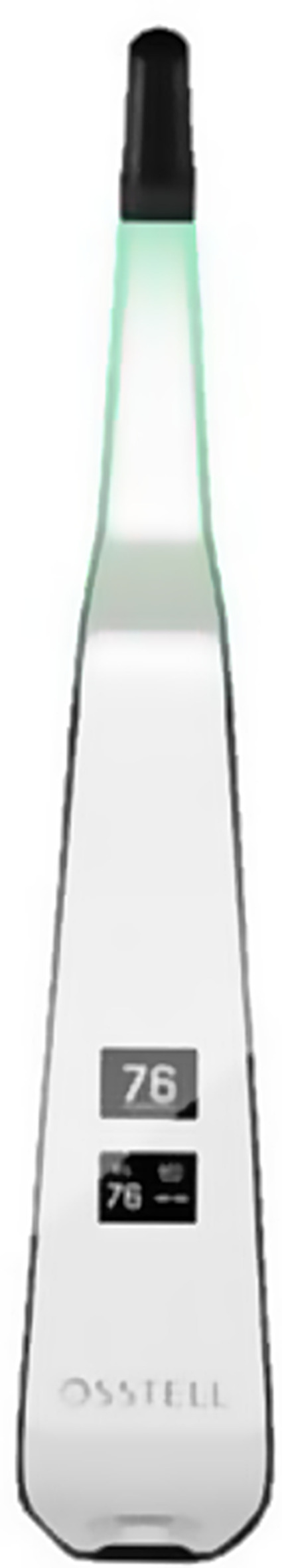
Ostell Beacon ^TM^ device

Implant stability was checked in both buccolingual as well as mesiodistal direction at the time of loading and after 1, 2, 3 and 4 months after implant placement with the device and RFA, shown in Fig. 3. The ISQ readings were entered into the specific patient data collection form.

The commercially available device was originally designed to assess the stability of endosseous implants and had SmartPegs ^**TM**^ for the same. As orthodontic buccal shelf implants possess a smaller dimension and a different design than endosseous implants, a customized smart peg was fabricated to assess their stability.

**Fig. 4:**
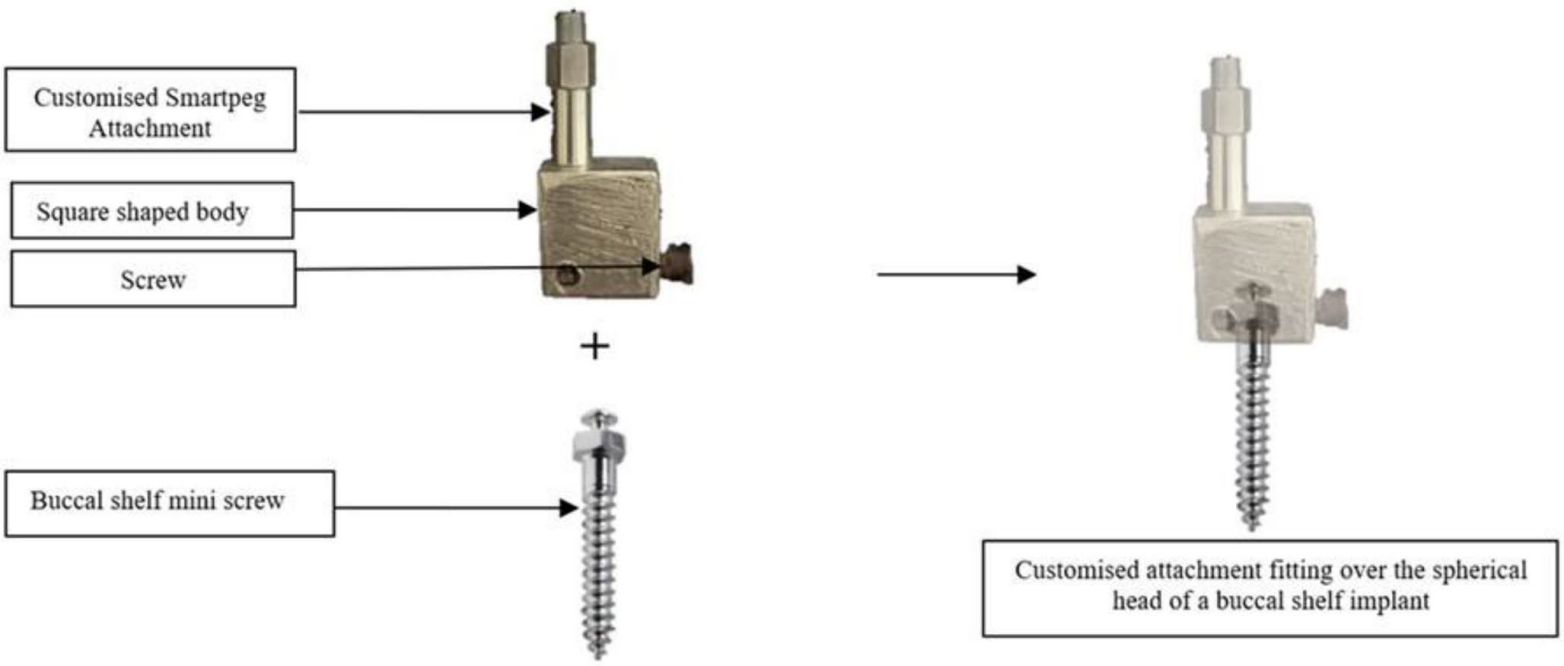
Schematic representation of the custom attachment and the buccal shelf bone screw

The custom-fabricated magnetic attachment (SmartPeg) was attached to the buccal shelf implant (Fig. 4). It was square and was made up of aluminium which is also the core material used in the manufacturing of commercially available SmartPeg ™. Aluminium is a good electrical and thermal conductor, nontoxic in nature, corrosion resistant, and easily formable. It also consisted of a zinc-coated magnet placed on top of it.

The attachment was screwed onto the spherical head of the buccal shelf implant with the help of a small external screw.

**Fig. 5:**
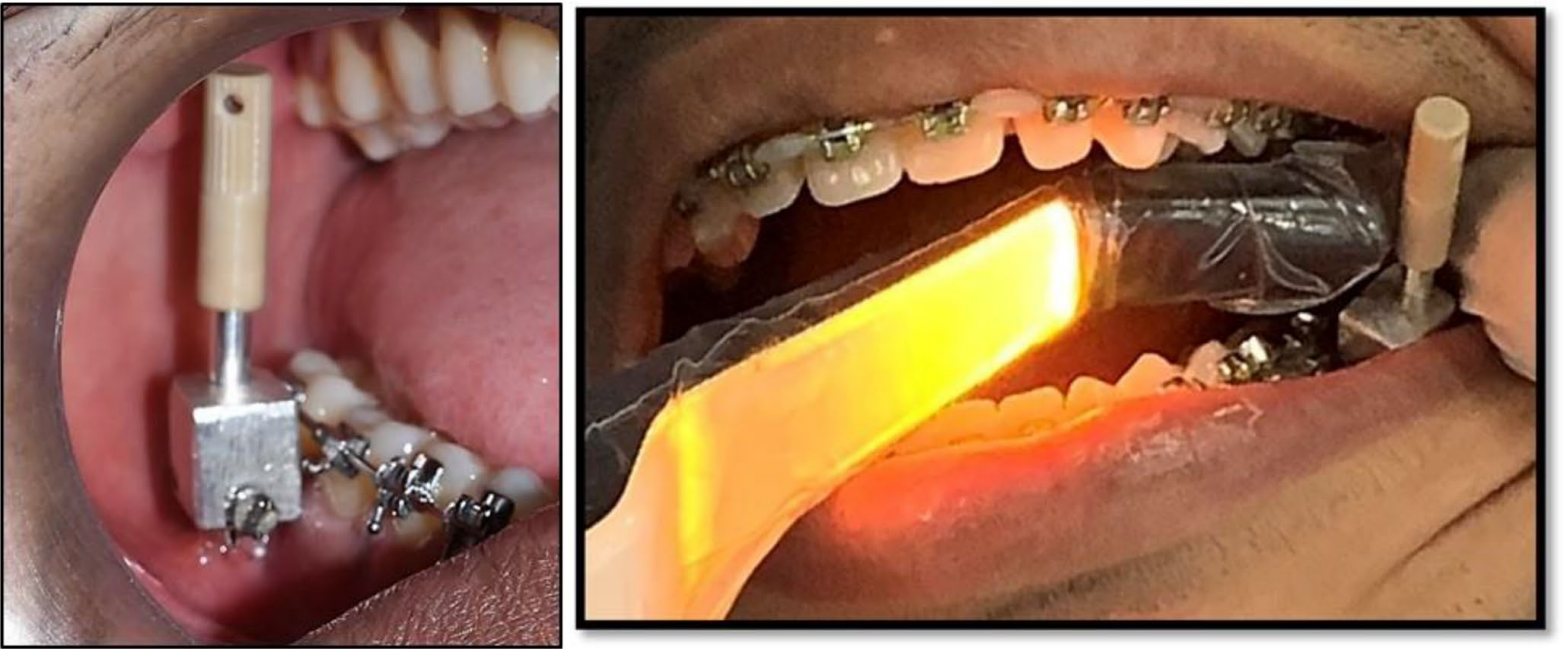
Intraoral placement of the customized Smartpeg attachment with Ostell device

When the magnet was brought close to the transducer probe, it was triggered by a magnetic pulse which was created. An audible sound was produced by the instrument upon capturing the response signal from the probe. The ISQ value, which ranged from 1 to 100, was then displayed, with 100 denoting the maximum stability (Fig. 5).

**Fig. 6:**
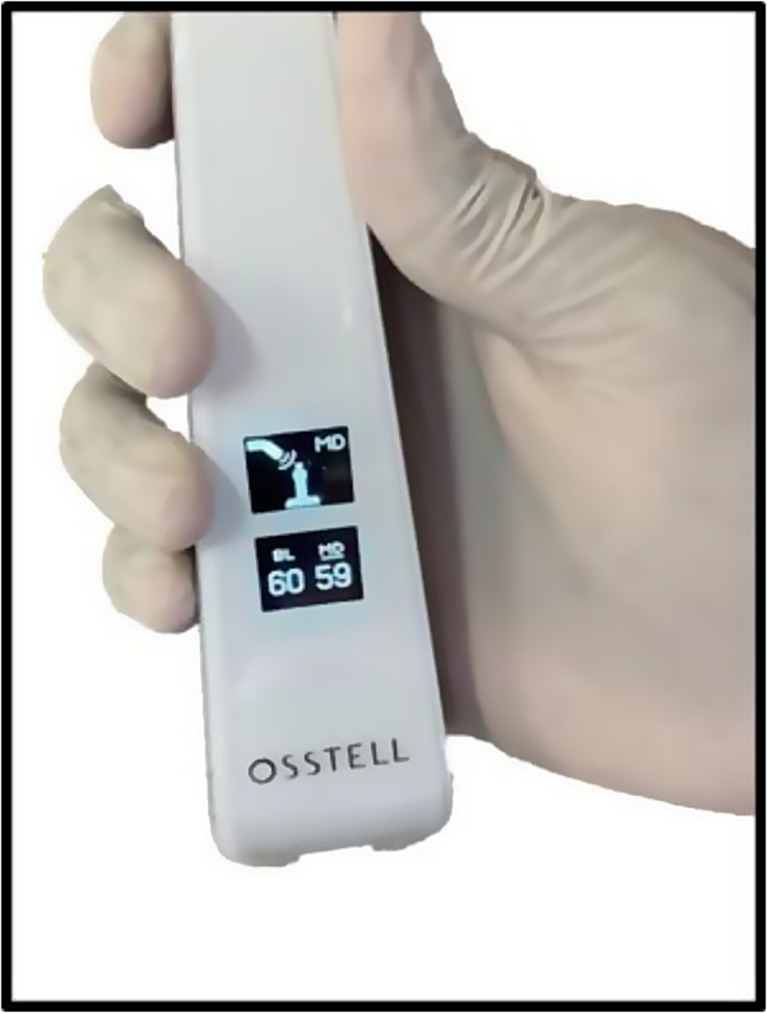
The ISQ readings displayed by the Osstell Beacon ^TM^ device in both buccolingual and mesiodistal directions

Figure 6 depicts the Osstell ^TM^ ISQ scale with readings of less than 60 indicating low stability, 60–69 indicating medium stability, and more than 70 indicating high stability.

### Statistical analysis

Version 20 of the Statistical Package for the Social Sciences (SPSS) was used to compile and enter the data. Using the relevant tables and figures, the results were expressed as proportion and summary measures (median with IQR). Friedman’s Two-Way Analysis of Variance and the Wilcoxon signed rank test were utilized for the intergroup comparison. A statistically significant result was defined as one with a p-value of less than 0.05. Pearson regression correlation analysis was used to compare the ISQ readings between both the groups.

**Fig. 7:**
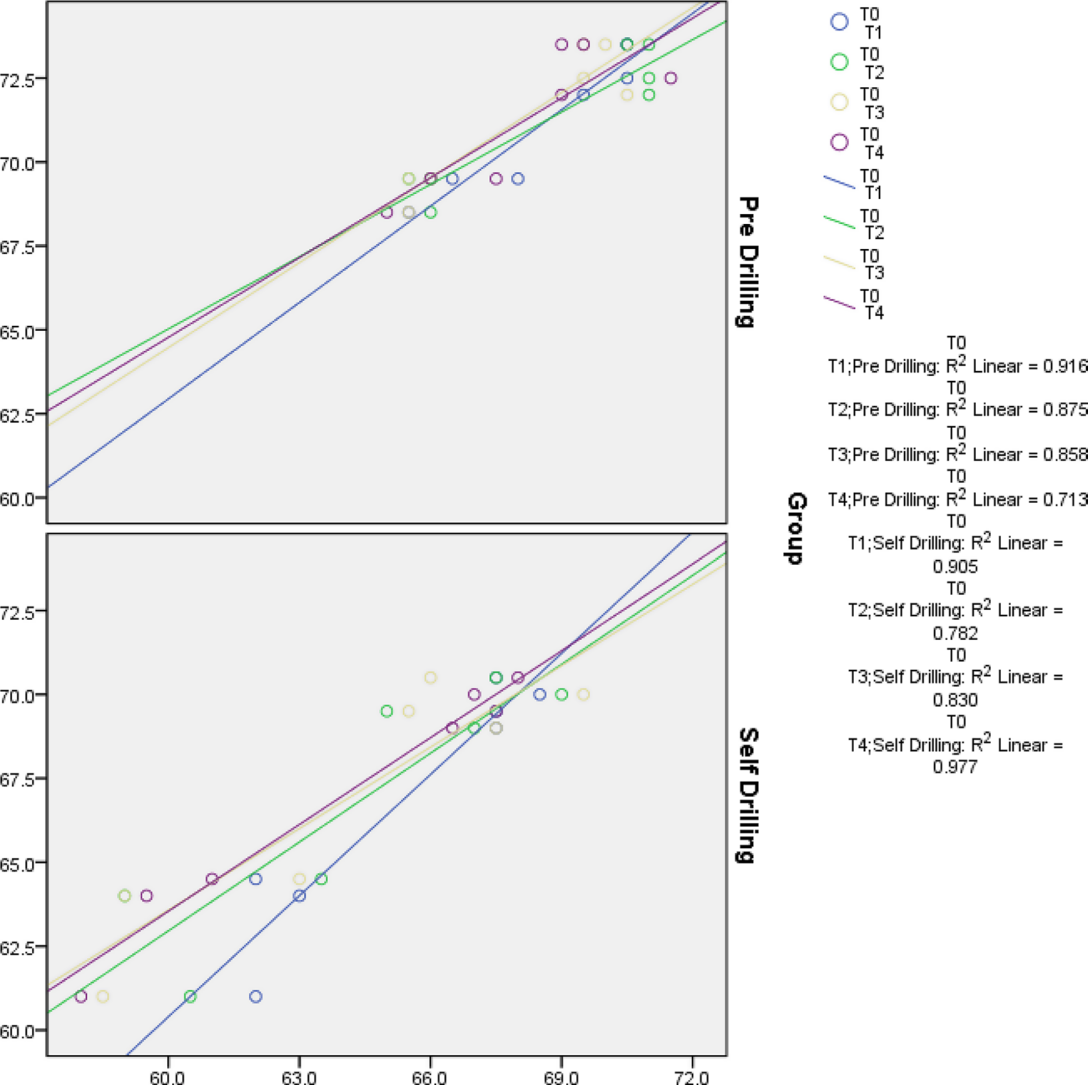
Pearson’s regression graph depicting the ISQ readings in both groups

## Results

The total sample of 14 buccal shelf implants was divided into two subgroups of 7 each. 7 buccal shelf bone screws were placed using the pre-drilling protocol and the other 7 were placed using the self-drilling protocol in a split-mouth study design. Table 1 depicts the ISQ readings in the two groups at various time intervals. The ISQ readings in GROUP 1 were 72.0 (T1), 70.0(T2), 70.0(T3), and 69.0(T4).

**Table 1:** ISQ readings in both groups at each time interval

	T0	T1	T2	T3	T4
Self- drilling	69.0	68.0	65.0	66.0	66.0
Pre-drilling	72.0	70.0	70.0	70.0	69.0

Table 2 shows the comparison of the ISQ values between pre-drilling and self-drilling groups at T0 i.e., at the time of placement of the screw. The median ISQ value for the pre-drilling group was 72.0 and for the self-drilling group was 67.0. The result indicates a statistically significant difference between the implant stability readings between the two groups indicating significantly higher implant stability in the pre- drilling group, when compared to the self-drilling group. (p value = 0.018).

**Table 2:** Comparison of the ISQ values between the pre-drilling and self-drilling groups at T0

	SAMPLE SIZE	MEDIAN	INTERQUARTILE RANGE (IQR)	*P* VALUE
Pre-drilling group	**7**	**72.0**	**70.0–74.0**	0.018*
Self-drilling group	**7**	**67.0**	**64.0–70.0**	0.018*

The comparison of the ISQ values between two groups between T1-T4 is shown in Table 3. The median ISQ value for the pre-drilling group from T1-T4 is 70.0 and for the self-drilling group is 67.0. The result indicates a statistically significant difference between the implant stability readings between the two groups from T1-T4 indicating significantly higher secondary implant stability when compared to the self-drilling group. (p value = 0.018*)

**Table 3:** Comparison of the ISQ values between the groups during T1-T4 table 3:

	SAMPLE SIZE	MEDIAN	INTERQUARTILE RANGE (IQR)	*P* VALUE
Pre-drilling group	**7**	**70.0**	**66.0–70.0**	0.018*
Self-drilling group	**7**	**67.0**	**59.5–68.0**	0.018*

Table 4: Change in ISQ readings amongst the periods assessed in the study for both groups There was a statistically significant change in the pre-drilling group (Group 1) from the time of placement to the first month (T0 to T1), but the ISQ readings remained stable thereafter in both groups. There was no significant difference in the ISQ readings between the first and the fourth month (T0 and T4).

**Table 4:** Change in ISQ readings amongst the periods assessed in the study for both groups

PERIOD	MEDIAN	*P* VALUE
PD0	-2.0	0.027*
PD1	0.0	0.317
PD2	0.0	0.666
PD3	0.0	0.336
PD4	0.0	0.470
SD0	-2.0	0.820
SD1	-1.0	0.820
SD2	0.0	0.820
SD2	0.0	0.820
SD4	-1.0	0.470

Pearson’s Regression correlation test was done to compare the inter – group ISQ readings at different time intervals. The results indicated a high degree of consistency in the measurements across different time points for both pre-drilling and self-drilling analyses. The correlations were generally very strong, with most P-values indicating statistical significance, highlighting the reliability of the measurements over time.

## Discussion

In the current era, temporary anchorage devices have gained tremendous attention and popularity due to their versatile applications in clinical orthodontics. Several sites have been used for temporary anchorage device insertion such as the palatal bone, infra zygomatic crest area, buccal cortical plate, the mandibular retromolar area, and the posterior alveolar process of the palate.

The mandibular buccal shelf has lately been proposed as a viable location for the insertion of extra-alveolar bone screws. It is located in front of the oblique line of the mandibular ramus, buccal to the roots of the first and second molars, bilaterally in the posterior region of the mandibular body [4].

When compared to traditional interradicular mini-screw insertion sites, this position provides significant clinical advantages. The orthodontist can position the screws parallel to the long axes of the molar roots since it extends buccally with a substantial amount of bone, which minimizes the chance of screw-to-root contact during anterior dental movements. Another significant benefit is a decreased chance of screw-to-root contact during insertion, which is one of the most frequent reasons for implant failure. Chang et al. in 2015, reported reduced failure rates in buccal shelf bone screws as compared to inter radicular screws [1].

Sufficient stability, both primary and secondary is essential for the longevity of an implant and successful conservation of anchorage. Numerous factors influence the stability of orthodontic bone screws which include angulation of the implant to the bone, site of implantation, degree of implant-to-bone contact, thickness, and mobility of soft tissues, insertion and removal torque and quality, implant site preparation, and quantity of cortical bone [10, 11]. 

According to Baumgaertel et al. [12–14], the most important factor which determines implant stability is insertion torque. It is the amount of torque applied during the insertion of an implant and is indicative of the resistance the implant encounters which is directly proportional to the amount of bone compression during placement. It increases with greater cortical bone thickness and serves as an indirect measure for the primary stability of the mini-implant [15]. They suggested that, for maximum stability, the insertion torque should ideally fall between 5 and 10 Ncm. This is achievable with a cortical bone thickness of roughly 0.5 to 1 mm according to a study done by Wilmes et al. [16] It has been observed that the insertion torque is considerably higher in areas with thick, dense cortical bone like the mandibular buccal shelf, median upper alveolar process, and around the mid palatal suture. This negatively impacts bone remodelling and results in a lack of secondary stability, bringing down the overall success rate of the implant to 60.9%, along with an increased risk of implant fracture. Reduced initial insertion torque during bone screw installation is therefore, the goal [17].

The technique used for implant placement significantly affects bone compression and insertion torque. Two protocols are widely used for the placement of buccal shelf bone screws. The first is a self-drilling protocol wherein the screw is placed with the aid of the sharp cutting edges of the screw itself, without a punch cut or a pilot hole. The second approach is a pre-drilling technique wherein an initial tissue punch cut is made, followed by a pilot hole at the site of implant placement. This weakens the adjacent cortical one and reduces the resistance experienced during placement [18]. Clinicians are often faced with the dilemma of which protocol to use to ensure maximum stability and successful treatment outcomes. Keeping this in mind, the present study was designed to evaluate and compare the stability of buccal shelf screws inserted using both the above-mentioned protocols.

There are various methods used to assess implant stability such as the pull-out test, insertion torque analysis, removal torque assessment etc. The disadvantage of these techniques is that they are only valid at the time of placement and are not reproducible [19].

A newer technique known as the Resonance Frequency Analysis, which is based on the tuning fork principle, was employed in the present study with the help of the Osstell Beacon ^TM^ device. The device came with attachments compatible with conventional prosthetic implants. However, orthodontic implants differ significantly from dental implants in terms of their design, surface characteristics and size. To ensure a strong connection between the buccal shelf implant and the transducer, a specially designed smart peg was created, with properties like the commercially available Smartpeg ™. Each implant was fitted with a sterile, disposable Smartpeg and the frequency with the strongest vibration was recorded as the resonance frequency. This measurement is non-invasive, reproducible and swift. The outcome is displayed as a value in the range of 1 to 100; >70 ISQ is regarded as high stability, 60–69 as medium stability, and < 60 ISQ as low stability [20].

In the present study, we assessed and compared the stability of the bone screws at four different periods between the two placement protocols: pre-drilling and self-drilling. Excellent stability was observed in both groups across the time periods assessed. However, a higher Implant Stability Quotient (ISQ) was observed in the pre-drilling group as compared to the self-drilling group. This can be attributed to the fact that an initial pre-drill weakens the adjacent cortical bone, thereby reducing the insertion torque which results in increased stability.

According to the current investigation, the buccal shelf bone screw implantation resulted in maximum stability at the time of placement, which then declined. The relaxing of the surrounding hard tissue brought on by bone resorption as a result of osteoclast activity during the early healing phase may explain this phenomenon. This corroborates the theory that, as previously demonstrated for conventional dental implants, primary stability is most potent just after placement and diminishes over time. The physiological processes that are taking place in and around the implant can explain this. After two hours of implant placement, neutrophils, macrophages, and erythrocytes combine to form a fibrin network. On the fourth day following implant placement, osteoclasts and mesenchymal cells start to emerge and begin to eliminate any bone damage. As seen in the current study, this causes the stability to decline after the first month [21].

Deguchi et al. conducted another study in 2009 that evaluated the histological healing of the osseous tissue surrounding mini screws used as an orthodontic anchorage as well as the alteration in cortical bone thickness at 3, 6, and 12 weeks after the screws were inserted. After three weeks, it was found that, in comparison to the control, there was less cortical bone thickness in all regions of the jaw. Bone-implant contact revealed less bone in all areas surrounding the implant when compared to the maxilla. This showed that the mandible did not recover its cortical bone thickness even after three weeks of healing [22]. This confirmed the current study’s findings, which depicted that after the first month, ISQ measurements dropped.

This study shows that both the protocols result in a high degree of implant stability with a marginally higher level of stability with the pre-drilling method, over time. This outcome will help clinicians make informed decisions while choosing the best technique to place buccal shelf screws. Due to the novel design of the customized Smart peg attachment, further studies can be done to evaluate its efficacy for measuring the stability of the buccal shelf bone screws using a bigger sample size.

## Data Availability

No datasets were generated or analysed during the current study.
